# Physiotherapeutic treatment of scoliosis in Austria and in international comparison

**DOI:** 10.1186/1748-7161-9-S1-O65

**Published:** 2014-12-04

**Authors:** Petra Groebl, Esther Klissenbauer

**Affiliations:** 1 FH-JOANNEUM University of Applied Sciences, Graz, Austria; 2 Physio Austria, Professional Association Physiotherapy, Vienna, Austria

## Background

In December 2011 a new working-group “SCOLIOSIS” was founded as part of the professional association of physiotherapists in Austria (Physio Austria) to foster the work with patients suffering from scoliosis.

## Aim

In a first step this working-group aimed for an in-depth view on the current provision of physiotherapeutic care for patients, who suffer from scoliosis and to establish a national and international network of people who put their main emphasis on the treatment of scoliosis. Furthermore the collected data should help to answer necessary questions of health insurance companies or other interested parties with regard to the provision of physiotherapeutic care of patients with scoliosis.

## Design

Cross-sectional study.

A questionnaire with 14 (quantitative and qualitative) questions was compiled. The questionnaire included questions on patient characteristics, training requirements of physiotherapists, cooperation among different professions or the need of treatment opportunities (i.e. rehabilitation centres, holiday camps).

## Methods

Data for this cross-sectional study was collected only from physiotherapists. All physiotherapists were members of Physio Austria 2012 and/or members of SOSORT 2012. The questionnaires were sent by email.

## Results

Both groups, Austrians (n=752) and SOSORT members (n=52) put emphasis on high quality treatments. SOSORT members treat their patients with various Scoliosis concepts. In Austria physiotherapists prefer Schroth-therapy. In both groups physiotherapists see a need (n=342) for rehabilitation centers for scoliosis with intensive treatment as an in-patient as well as an out-patient. There is no significant difference in international comparison regarding the wish to work in an interdisciplinary team with orthopaedists, orthopaedic technicians and other physiotherapists whose main emphasis of their work is scoliosis.

## Conclusions

Apart from correcting inconvenient everyday postures, physiotherapists all over the world understand therapeutic exercises to be the key to a positive development of juvenile spines. Hence, individual correcting exercises on high standard are particularly important for them.
